# Radiogenomics: Hunting Down Liver Metastasis in Colorectal Cancer Patients

**DOI:** 10.3390/cancers13215547

**Published:** 2021-11-05

**Authors:** Carolina de la Pinta, María E. Castillo, Manuel Collado, Cristina Galindo-Pumariño, Cristina Peña

**Affiliations:** 1 Radiation Oncology Department, Ramón y Cajal University Hospital, IRYCIS, Alcalá University, 28034 Madrid, Spain; 2 Medical Oncology Department, Ramón y Cajal University Hospital, IRYCIS, Alcalá University, 28034 Madrid, Spain; marienz707.cs@gmail.com (M.E.C.); manualmansa98@gmail.com (M.C.); crisgpuma@gmail.com (C.G.-P.); 3 Centro de Investigación Biomédica en Red de Cancer (CIBERONC), 28029 Madrid, Spain

**Keywords:** colon cancer, liver metastasis, radiogenomics, radiomics, metastatic niche, early detection

## Abstract

**Simple Summary:**

Colorectal cancer (CRC) is the third leading cause of cancer and the second most deadly tumor type in the world. The liver is the most common site of metastasis in CRC patients. The conversion of new imaging biomarkers into diagnostic, prognostic and predictive signatures, by the development of radiomics and radiogenomics, is an important potential new tool for the clinical management of cancer patients. In this review, we assess the knowledge gained from radiomics and radiogenomics studies in liver metastatic colorectal cancer patients and their possible use for early diagnosis, response assessment and treatment decisions.

**Abstract:**

Radiomics is a developing new discipline that analyzes conventional medical images to extract quantifiable data that can be mined for new biomarkers that show the biology of pathological processes at microscopic levels. These data can be converted into image-based signatures to improve diagnostic, prognostic and predictive accuracy in cancer patients. The combination of radiomics and molecular data, called radiogenomics, has clear implications for cancer patients’ management. Though some studies have focused on radiogenomics signatures in hepatocellular carcinoma patients, only a few have examined colorectal cancer metastatic lesions in the liver. Moreover, the need to differentiate between liver lesions is fundamental for accurate diagnosis and treatment. In this review, we summarize the knowledge gained from radiomics and radiogenomics studies in hepatic metastatic colorectal cancer patients and their use in early diagnosis, response assessment and treatment decisions. We also investigate their value as possible prognostic biomarkers. In addition, the great potential of image mining to provide a comprehensive view of liver niche formation is examined thoroughly. Finally, new challenges and current limitations for the early detection of the liver premetastatic niche, based on radiomics and radiogenomics, are also discussed.

## 1. Introduction

Colorectal cancer (CRC) is the third leading cause of cancer and the second most deadly tumor type in the world [1]. The liver is the most common site of metastasis in CRC patients [2]. Approximately 50% of CRC patients will develop liver metastasis at some point during their disease course [3]. Prediction of the development of liver metastasis and the response to treatment or survival of these patients would help to improve therapeutic protocols. This is why several studies have looked at how mathematical models for diagnosis, prediction of response and survival in patients with metastatic CRC (mCRC) can be developed from radiomics and radiogenomics technology.

Radiomics is a developing discipline that analyzes and extracts data from medical images, including quantitative and qualitative characteristics invisible to the human eye. The development of this type of analysis requires the acquisition of images; the creation of datasets; the export of DICOM files; the identification of the relevant volume by automatic, semi-automatic or manual segmentation tools; the extraction and qualification of image features; the use of the data generated; the construction of a predictive model; and the validation of the models created. Morphological features obtained include volume, shape, 3D geometry, diameter, surface area, sphericity, location, vascularization and necrosis, among others. First-order statistics include mean, median, standard deviation (SD), kurtosis and entropy, among others. Second-order statistics include the ratio in an inter-voxel image, the co-occurrence matrix, matrix length and matrix size, among others. Higher or higher-order statistics include the relationship with neighboring voxels. For the prediction model, clinical, pathological and genomic relationships are established. Thus, radiogenomics allows the integration of radiomic findings and molecular alterations, facilitating precision medicine tools such as diagnosis, prognosis, prediction of response or recurrence and improved treatment selection. Several publications explain the use of radiomics and radiogenomics in primary liver tumors [4]. However, there are few studies of this discipline in liver metastasis.

In this study, we reviewed the knowledge gained from radiomics and radiogenomics studies in hepatic metastatic colorectal cancer patients and their possible use as clinical tools in colorectal cancer patients’ management. We also studied the chance to provide a comprehensive view of liver niche formation by radiomics and radiogenomics. Therefore, we searched in PubMed and MEDLINE for the following keywords: “radiogenomics liver metastases”, “radiogenomics liver cancer”, “Radiomics AND angiogenesis”, “Radiomics AND Immune surveillance”, “Radiomics AND Immune”, “Radiomics AND early cancer liver diagnostic” and “Radiomics AND early liver metastases diagnostic”.

## 2. Clinical Benefit of Radiogenomics in Metastatic Colorectal Cancer Patients

Numerous studies focused on radiomics and radiogenomics studies in hepatic metastatic colorectal cancer patients pointed out their use in early diagnosis, response assessment and treatment decisions (Table 1).

### 2.1. Early Diagnosis of Colorectal Cancer Metastasis

Computed tomography (CT) is the most common imaging test for studying CRC patients. However, its ability to detect liver metastases is limited. Magnetic resonance imaging (MRI) and tissue biopsy are used in selected cases, but these techniques delay patients’ diagnosis and treatment starting point. As radiomics could help in the diagnosis of liver metastasis by CT, in line with the data from some studies, Becker and collaborators investigated various texture features. These included a grey-level co-occurrence matrix, grey-level run-length matrix and grey level size-zone matrix. Interestingly, they found a correlation between these features and the occurrence of metastasis prior to their detection by conventional CT methods [5]. Taghavi et al. and Rao et al. designed a prediction model for the detection of metachronous metastasis [6,7]. Other authors analyzed MRI (T2 sequences) to extract radiomic features [8]. Generally, metastasis appears to be characterized by high entropy, heterogeneity and variance and may be explained by cell clones, necrosis and vascularization [29].

To diagnose hepatic lesions, differential diagnosis between different entities is required. Some authors have studied radiomic parameters in MRI tests to help to differentiate between tumor and non-tumor lesions [9,10]. Jansen and collaborators used contrast curve, grey-level histogram and grey-level co-occurrence matrix texture features in MRI images (DCE and T2 sequences), combined with clinical factors such as steatosis, cirrhosis and tumors of unknown origin. They classified lesions into five categories: adenoma, cyst, hemangioma, hepatic primary tumor and metastases of varying sensitivity and specificity (0.8/0.78, 0.93/0.93, 0.84/0.82, 0.73/0.56 and 0.62/0.77, respectively) [11], which was similar to the data of Gatos et al. [12]. In short, several authors agree that texture analysis can help to differentiate between liver metastases and other types of liver lesion [29].

### 2.2. Response Assessment and Treatment Decision Tool

The correct assessment of response in the treatment of CRC with liver metastasis is fundamental in defining the success or failure of treatment interventions. In addition, prediction of early response would improve treatment selection in these patients. In this context, radiomics and radiogenomics could be very useful.

In the Taghavi et al. study, progression after radiofrequency was assessed with 1,593 radiomic parameters extracted from each lesion [13]. Three prediction models were constructed: one with radiomic parameters, one with clinical parameters and one with a combination of radiomic and clinical parameters. This last model had the highest predictive value. Staal et al. extracted radiomic parameters eight weeks after radiofrequency treatment in a 10 mm ring from the periablation zone and from the ablation zone on CT in the portal venous phase. The combination of skewness, uniformity and mean in the periablation ring were predictors of progression. Again, predictive ability improved when clinical parameters were combined [14]. Another study evaluated response after radioembolization with Itrio 90: texture parameters were able to detect relapses 3.5 months earlier than RECIST criteria [15]. However, not all studies have found statistical differences [16].

The assessment and prediction of response to systemic neoadjuvant treatment is essential, as this avoids delay in surgery or in the selection of alternative treatments if patients do not respond. In addition, in unresectable patients, predicting the response to treatment can avoid ineffective treatment regimens and major side effects.

In patients treated with FOLFOX or FOLFIRI, low skewness was associated with a high response rate to chemotherapy, validated in an external cohort [17]. In the evaluation of response with dual anti-Her2 treatment, another study identified heterogeneity features related to treatment response, although the results need to be validated, as the study authors themselves affirmed [18]. High entropy and low homogeneity after chemotherapy were associated with earlier response prediction than RECIST [19,20,21,22]. These data suggest that texture may be a predictor of response in patients receiving chemotherapy.

### 2.3. Radiomics as a Prognostic Tool

Radiomics and radiogenomics could also become a prognostic assessment tool in mCRC patients, as some authors have suggested.

An association between entropy and prognosis has been demonstrated [19,20,22]. Homogeneity in the texture of healthy liver tissue is predictive of worse survival [23,24]. Andersen et al. described, with CT images, an association between homogeneity parameters and worse overall survival (OS) [20]. However, Rahmim and collaborators, in a multivariate analysis, showed radiomic parameters of heterogeneity on FDG PET as predictors of lower OS [25]. Lubner et al. reported that the degree of skewness was inversely related to mutations in KRAS and that entropy was associated with OS [22]. In the same study, the authors demonstrated the association of lower entropy, SD and high mean positive pixels with tumor grade in CT images, validating the results. In addition to the survival advantages of some imaging parameters, the possibility of stratifying patients for recurrence in liver remnants has been shown [23]. Ravanelli et al. related high CT uniformity and low OS and PFS in patients with CRC and liver metastasis [28].

Some studies analyzed radiomic parameters for survival prediction with various chemotherapy schedules. In one study, radiomic parameters associated with patients treated with FOLFIRI with or without cetuximab were found to be predictors of sensitivity and were associated with OS [26]. In the combination of first-line FOLFIRI and bevacizumab [27], the decrease in the sum of lesions, the decrease in kurtosis and the high density of DLL were predictors of OS. These findings were confirmed in an external cohort, but the morphological response was not associated with OS, which cast doubt on the usefulness of RECIST.

## 3. Liver Premetastatic Niche Formation in CRC Patients

Invasion and tumor cell growth are necessary for metastasis formation, but only 0.01% of circulating tumor cells are able to develop distant metastatic nodes [30]. The liver is the most common site for metastasis in CRC patients [2], due in part to anatomical distribution since the portal vein and hepatic artery supply blood to the liver and, in part, because cancer cells disseminating from the colon easily access the liver through the portal vein [31].

**Figure 1:**
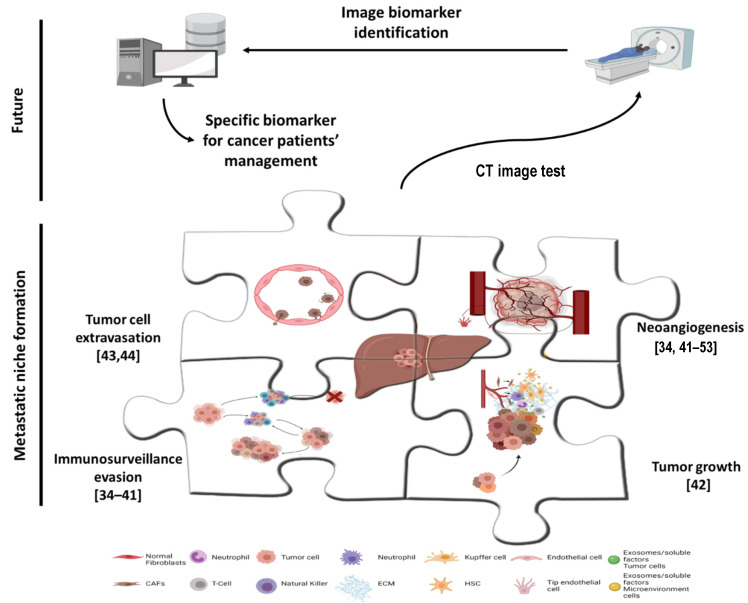
Hunting down liver metastasis in colorectal cancer patients by radiogenomics. The figure shows a comprehensive view of metastatic niche formation phases. Reasonably, the different phases of metastatic niche formation (each one represented by a puzzle piece in the figure) could be detected by radiogenomics approaches in the near future. The references included in the figure show related manuscript with information about tumor cell extravasation [34,35], neoangiogenesis [34,35,36,37,38,39,40,41,42,43,44,45,46,47], immunosurveillance evasion [44,47,48,49,50,51,52,53] and tumor growth [41] that could be theoretically translated to metastatic niche detection. Created with BioRender.com.

Tumor-derived factors, including pro-angiogenic and pro-inflammatory factors, are released from the primary tumor to prepare distant metastatic niches [32,33]. These factors promote the recruitment into the hepatic pre-metastatic niche of different microenvironment cells, such as Kupffer cells, hepatic stellate cells, myeloid-derived suppressor cells and neutrophils, all of which play a key role in niche generation [31]. This process can be divided into three different phases: extravasation and angiogenic process, immune surveillance evasion and organotropism and tumor growth. Imaging could provide a comprehensive view of these niche formation phases, thus increasing the early detection of liver metastasis in CRC patients. It might even be possible to detect early changes in the metastatic niche that are not captured by standard clinical imaging techniques during the follow-up of patients (Figure 1). Such changes could lead to the adjustment of therapy towards more aggressive treatments that might disrupt metastatic growth.

### 3.1. Extravasation and Angiogenic Process

Primary tumor cells migrate to blood vessels by means of epithelial–mesenchymal transition, when they lose their epithelial properties and move across the extracellular matrix [54,55]. Extravasation in the liver is also a complex process with many components involved [30,56,57]. The liver is a highly irrigated organ, which is a clear advantage for tumor colonization, but neoangiogenesis is needed to maintain the high nutrient and oxygen demand of tumor cells [57,58,59]. Evidence suggests the usefulness of radiomics or radiogenomics to detect epithelial–mesenchymal transition, vascular invasion, neoangiogenesis and microvascular density. Xing Liu and collaborators established a contrasted enhancement-related gene expression signature by combining classic molecular–pathological biomarkers, whole-genome transcriptome sequencing, clinical characteristics, radiological manifestations and radiomics. The authors analyzed the data from 155 patients with anaplastic gliomas and found that identifying the texture features of radiomics by measuring the inhomogeneity of image patterns may reflect the neoangiogenesis and epithelial–mesenchymal transition of the tumor [34].

Microvascular invasion is an independent prognostic factor for the overall survival of hepatocellular carcinoma patients. However, it is not possible to analyze this parameter prior to the pathological analysis of tumor tissue. Thus, new biomarkers for the early detection of microvascular invasion are needed urgently. Liu P and collaborators showed the role of radiogenomic analysis in determining microvascular invasion in preoperative patients [36]. Moreover, another study corroborated the prognostic value of these radiogenomic biomarkers of microvascular invasion in hepatocellular carcinoma to predict patients’ recurrence and survival [37]. The histopathological growth patterns in colorectal liver metastasis include desmoplastic, pushing and replacement patterns and two rarer histopathological growth patterns. Differences in microenvironments’ heterogeneity involve response to treatments and patient survival. Angiogenesis sprouting and microvascular invasion are the two principal components defining the histological growth patterns [38,39]. Yuqi Han and collaborators developed an MRI-based radiomic model to predict the predominant histopathological growth patterns of colorectal liver metastasis as a potential biomarker for clinical treatment [60].

Other authors have described related imaging biomarkers to identify angiogenesis in brain tumors. For instance, aggressive biological processes of cell adhesion and angiogenesis were enriched in glioblastoma patients with poor overall survival [40]. Moreover, in glioblastoma patients, radiogenomics analysis showed a radiomic risk score associated with cell differentiation, cell adhesion and angiogenesis, which contributed to chemoresistance [35]. Similarly, in lower-grade glioma patients, a radiogenomics study revealed a prognostic radiomic signature as a biological surrogate, such as hypoxia, angiogenesis, apoptosis and cell proliferation, providing prognostic information for these patients [41]. Radiomic features could also reflect the angiogenesis status and microvascular density in bladder urothelial carcinoma and in clear-cell renal-cell carcinoma [42,43]. Radiomic parameters also predict microvascular density and angiogenesis in breast cancer [44,45,46]. In the study by Dooman Arefan and collaborators, a set of radiomic features identified the heterogeneity of tumor microenvironment cells, with an abundance of fibroblasts and the presence of endothelial and immune cells [44].

### 3.2. Immune Surveillance Evasion

After the arrival of the colon cancer cell to the liver, and when cells gain access to a blood supply, they proliferate to expand the metastatic niche. However, the activation of cytolytic T-cells, due to the presence of tumor cells, can abrogate tumor growth [61]. Thus, tumor cells can evade the cytotoxic T-cell response via the expression of co-inhibitory molecules such as CTLA-4 or PD-1 and the promotion of immune surveillance evasion [31]. Moreover, the recruitment to the metastatic niche of different immune cells, such as immunosuppressive lymphoid and myeloid subsets, enhances the tumor’s immune tolerance, which allows the tumor to grow [31]. Several authors described the use of radiomics or radiogenomics to determine the presence and the amount of immune cells in tumor tissue. For instance, Seung Hyuck Jeon and collaborators described a radiomic signature that predicts CD8+ tumor infiltration lymphocyte alterations and suggested its clinical utility to evaluate tumor immune status after chemoradiotherapy for rectal cancer patients [48]. Moreover, calculation of CD8 infiltration by a radiogenomics signature using CT images and RNA sequencing data was proposed, in order to predict the immune phenotype of advanced solid malignant tumors and clinical outcomes of immunotherapy-treated patients [49]. Similarly, PD-L1-positive and -negative non-small-cell lung cancer patients could be determined by a deeply learned score derived from ^18^F-FDG-PET/CT images. This score also predicts patients’ survival and could be used to guide individual pre-therapy decisions [50]. MRI radiomic features can also determine PD-1/PD-L1 expression and prognosis in intrahepatic cholangiocarcinoma patients [51].

In an interesting study by Yunfang Yu and collaborators, the association of a multiomic signature based on magnetic resonance imaging, radiomic features and tumor microenvironment characteristics, including immune cells, was analyzed. Key radiomic features were associated with various immune cells, including M0 macrophages, B-naïve cells and neutrophils, and could predict preoperative axillary lymph node metastasis in breast cancer patients, supporting surgical decisions [52].

Low and high tumor-associated macrophages can also be differentiated by nanoradiomic analysis, which reveals texture differences, unlike conventional image-derived tumor metrics. The latter were unable to differentiate tumors with varying TAM burdens [53]. Specific gene expression sets associated with immune cells and angiogenesis can also be identified by several different radiomic features in non-small-cell lung cancer patients [39].

### 3.3. Organotropism and Tumor Growth

The tumor and distal organs participate in cross-talk by chemokine secretion that conditions the metastatic niche and colon tumor cells’ organotropism to liver invasion. The interplay between different chemokines and receptors, such as CCL20, CCR6, CXCR4 [30,62] and other secreted proteins, such as carcinoembryonic antigen [63], ostopontin [59] and integrins including α6β1, α6β4 and/or α2β1 [59], is involved in the retention of metastatic cells in the liver, as well as in the preparation of the liver environment niche to allow colon tumor cells’ survival and growth. No specific radiomics or radiogenomics studies have evaluated the role of radiomic features with these factors. However, it is reasonable to suggest that specific radiogenomics studies to correlate these factors with radiomic features will offer data that would easily determine the expression of these metastatic growth factors by new image biomarkers.

## 4. Future Perspectives and Challenges for Early Detection of Liver Premetastatic Niche, Based on Radiogenomic Approaches in Colorectal Cancer Patients

As stated above, the main cause of CRC mortality is metastasis, which is most common in the liver. Thus, the prevention of recurrence and its early detection in colon cancer patients are the main goals in clinical practice to improve patients’ survival. To attempt to achieve these goals, treatment decisions are taken on the basis of the usual histological and clinical parameters. However, these do not accurately predict the appearance of tumor metastasis. In fact, adjuvant chemotherapy in stage III patients clearly benefits patients, but the theoretically beneficial effects in stage II patients are not clear [64]. Moreover, since oxaliplatin is associated with cumulative neurotoxicity, new data support the advantage of only 3 months of treatment in those patients with a supposed low risk of recurrence [65].

The involvement of the microenvironment in distal metastatic niche growth is widely accepted. In fact, the “seed and soil” hypothesis, put forward by Paget et al., suggested that tumor cells (*seeds*) travel to distant sites (*soil*), where the tumor microenvironment is favorable to colonization [57,66]. However, the current imaging approaches, used daily in the clinical management of cancer patients, do not reflect numerous microenvironmental factors, such as angiogenesis, immune cell landscape and stromal density, that might determine intra-tumor heterogeneity and thus patient survival. Now, though, radiomic imaging analysis offers the chance to determine these microenvironment events in different pathologies. Moreover, radiogenomics also supports the possibility of determining the link between various biomarkers and the biological heterogeneity of tumors, in order to obtain information about gene expression, signaling pathway activity and tumor microenvironment features. Taking into account both this scenario and the data given in this review, it is reasonable to assume that several tumor microenvironment deviations, needed for metastasis growth, could be detected by the identification of early signs of hepatic metastatic niche formation and modification. This theoretical framework would help to prevent the appearance of metastasis by supporting aggressive treatments in patients at high risk of recurrence, but would avoid these destructive therapies and their important secondary side effects in patients with low recurrence risk. In addition, early changes in the metastatic niche, which are not captured in standard clinical imaging techniques, could be detected during the follow-up of patients. Therapy could be adjusted towards more aggressive treatments or local radical treatments of the metastatic niche, such as stereotactic body radiotherapy (SBRT) to disrupt metastatic growth, could be administered. Ultimately, radiomics research will identify new prognostic biomarkers for setting up tailored and dynamic therapies based on the molecular characteristics of colon tumors, to prevent liver metastasis growth and thus improve patients’ survival. Following this idea, Marjaneh Taghavi and collaborators analyzed retrospectively the primary staging portal venous phase CT of 91 CRC patients, who were divided into two groups: patients without liver metastasis (at primary stage or during the 24 months following diagnosis) and patients without liver metastasis at diagnosis but who developed liver metastasis in the 24 months after diagnosis. The authors described a machine learning-based radiomics analysis of routine clinical CT imaging and provided valuable biomarkers to identify high-risk liver metastasis from CRC at primary staging [6]. In line with these data, John M Creasy and collaborators studied 120 stage II/III colon cancer patients grouped by liver recurrence, extrahepatic recurrence or no evidence of disease at 5 years. The liver parenchyma images were studied by radiomic techniques. Their data showed CT radiomics as a promising tool to identify those patients at high risk of developing liver metastasis [67]. Another approach by Francesco Fiz and colleagues focused on the radiomic features of the tumor, peritumoral tissue and non-tumoral parenchyma in liver sections from colorectal cancer metastasis. Interestingly, their radiomics analysis found modifications of the peritumoral tissue similar to those observed in the tumor, although the radiological view had shown that this peritumoral tissue was the same as the non-tumoral liver parenchyma. Moreover, texture differences identified the peritumoral microenvironment as a separate entity from the normal parenchyma [68].

Another important way to develop radiomics field research is to look at imaging tests that are routinely used in current clinical practice for colon cancer diagnosis and patient follow-up. The development of mathematical models based on these imaging tests could improve the clinical management of patients at no additional cost, thus promoting personalized medicine in a sustainable and efficient way within the National Health System.

## 5. Technical and Clinical Limitations of Radiomics

Although there are not yet many data, these important findings confirm the capacity of radiomics to detect invisible-to-the-eye features of normal liver parenchyma that are related to metastatic niche formation. However, many limitations of radiomics and radiogenomics studies make it difficult to standardize this imaging technology in oncology clinical practice. Radiomics and radiogenomics are developing disciplines with important limitations that need to be taken into account.

Perhaps the most important limitation is the heterogeneity of software analysis in different studies, together with the variety of imaging devices in different hospitals. This clearly hinders the interpretation of different data for meta-analysis and multicenter studies. Lesion segmentation, one of the first steps in radiomics analysis, is very important and may affect results [69]. However, there is no agreement as to the optimal segmentation algorithm. Some believe that manual segmentation is better and more realistic, but others support automatic segmentation to avoid inter-observer variability. Semi-automatic segmentation could be a good option, but this has not been defined. Moreover, the impact of CT contrast administration and the different acquisition protocols has not been widely studied, which means that there is no clear evidence of whether a pre-contrast or contrast image dataset is better in a radiomics study. Similarly, reliable cut-off values are also difficult to determine. Imaging units are not the same in all centers, which can be especially important, for example, in MRI from 1.5 to 3 Teslas [16]. Image acquisition protocols vary from institution to institution and make validation more complex [29]. Due to these limitations, most studies have a new constraint based on the number of analyzed patients and a lack of independent validation cohorts. Since it is difficult to find homologous patient series based on similar image datasets, most analyses are based on retrospective studies of the patients. In addition, validation between series should be homogeneous, as different treatments may affect the comparability of results [6,70]. In addition, most of the studies examine only one geographic region.

Furthermore, treatment response assessment studies compare their results with RECIST, but it is known that RECIST also has limitations in response assessment [29].

Another important limitation in this research field is the lack of accuracy of “radiomics” and “radiogenomics” terminology. Many studies are classified as radiomics analysis, although only texture features (entropy, uniformity, kurtosis, skewness, standard deviation) are analyzed. In addition, genomic studies are sometimes associated with radiotherapy effects or imaging test changes. Obviously, although studies of standard image characteristics and associations are highly relevant in cancer research and are very useful during mCRC diagnosis and patient follow-up, these studies do not qualify as “radiomics” and “radiogenomics” studies. Moreover, they do not have the robust potential of an imaging biomarker roadmap, which radiomics or radiogenomics fields do have. This terminological confusion could create misunderstanding in the literature for readers and researchers.

The imaging biomarker standardization initiative (IBSI) has been proposed by Zwanenburg et al. [71]. This initiative includes regulation and consensus on image post-processing, segmentation, interpolation, intensity conversion, feature extraction and guidelines providing standardized definitions and validated reference values that facilitate their clinical use.

## 6. Conclusions: Radiomics Data Derived from Image Tests Are Postulated as Clear Surrogates

Radiogenomics, the computer extraction of mineable data from image tests together with the integration of genomic elements, offers an opportunity to deepen our understanding of the heterogeneity of the tumor microenvironment, specific tumor mutation and the main tumor-activated pathways. In short, it can generate promising radiomic signatures from entire organs, which may serve as good surrogate biomarkers to grasp, in a non-invasive and extremely personal way, “what is going on in the tumor” and, in the case of CRC liver metastasis, to decode early metastatic niche phenotypes.

Radiomics and radiogenomics are very young research fields. These tools have great potential for clinical use in the context of personalized medicine. Their utility has been demonstrated in early diagnosis, differential diagnosis, treatment selection and patient prognosis. However, multiple limitations have to be overcome before this technology can be translated to the clinical management of cancer patients. Nevertheless, they undoubtedly show tremendous potential for improving our knowledge and developing new clinical tools, based on the application of computer techniques and data processing. These tools can be used to plan the treatment of cancer patients early, dynamically and individually, i.e., not simply treating the disease but attempting to administer personally tailored medicine.

## Figures and Tables

**Table 1:** Clinical benefits of radiomic and radiogenomics in CRC liver metastatic patients.

Study	Design	Imaging Modalities	Sample Size	Study Cohorts and Validation	Tools for Radiomics Calculations	Statistical Model Construction
Early diagnosis of colorectal cancer metastasis						
Becker et al., 2018 [5]	Preclinical	MRI	8 male mice	One cohort	MATLAB routine	Linear regression model, Pearson correlation test and hierarchical cluster analysis
Taghavi et al., 2021 [6]	Retrospective	CT	91 CRC without LM at diagnosis	Two cohorts. Patients with metastases in follow-up of ≥24 months (*n* = 67); and patients who developed metachronous liver metastases <24 months (*n* = 24). No validation	Philips Intellispace Portal software and PyRadiomics	Kruskal–Wallis test, inter-correlated features and Bayesian-optimized random forest was used for prediction models.
Rao et al., 2014 [7]	Retrospective	CT	29 CRC patients	Three cohorts. Patients without LM (*n* = 15), with synchronous LM (*n* = 10) and metachronous LM within 18 months following primary staging (*n* = 4). No validation	MATLAB routine	Student’s *t* test or Mann–Whitney *U* test. ROC analyses to determine the potential diagnostic performance of the respective texture parameters for diagnosing the presence of metastatic disease.
Liang et al., 2019 [8]	Retrospective	MRI	108 rectal cancer patients	Two cohorts. 54 patients with LM and 54 without LM. The results of the one-round cross-validation were stabilized and representative.	Python in Anaconda3 platform with Scikit-learn and Matplotlib packages.	Models were evaluated with indicators of accuracy, sensitivity, specificity and AUC, and compared by DeLong test.
Oyama et al., 2019 [9]	Retrospective	MRI	150 liver tumors. 50 HCC, 50 LM and 50 HHs in 37, 23 and 33 patients	One cohort.	MATLAB Image Processing Toolbox, Signal Processing Toolbox, Statistics and Machine Learning Toolbox, and Wavelet Toolbox	Two machine learning models: a logistic classifier model with an elastic net penalty and extreme gradient boosting (XGBoost)
Li et al., 2017 [10]	Retrospective	MRI	162 patients	Three cohorts. HHs (*n* = 55), LM (*n* = 67) and HCC (*n* = 40). The test datasets validated the reliability of the models	R software (R Core Team, Vienna, Austria) and MATLAB R2013b (Mathworks, Natick, MA, USA)	Kruskal–Walls test, ROC curve and AUC analysis to differentiate three subtypes. K-nearest neighbor classifier model, back-propagation artificial neural network classifier model, support vector machine and logistic regression were used for improving accuracy for classifier.
Jansen et al., 2019 [11]	Retrospective	MRI	95 patients with 125 benign lesions and 88 malignant lesions	Two cohorts, benign and malignant lesions. 40 adenomas, 29 cysts and 56 HHs; and 30 HCC and 58 LM. Optimization process using cross-validation.	-	ANOVA F-score was selected and fed into an extremely randomized trees classifier and ROC curve analysis.
Gatos et al., 2017 [12]	Retrospective	MRI	71 FLLs. 30 benign lesions and 41 malignant lesions	Three cohorts. 30 benign lesions, 19 HCC and 22 LM. No validation	-	Probabilistic Neural Network (PNN) model evaluation was performed using the leave-one-out (LOO) method and receiver operating characteristic (ROC) curve analysis. Multilinear regression analysis.
Response assessment and treatment decision tool						
Taghavi et al., 2021 [13]	Retrospective	CT	90 CRC patients with 140 LM treated by ablation	Two cohorts. Training (*n* = 63 patients/*n* = 94 lesions) and validation (*n* = 27 patients/*n* = 46 lesions) cohort. Each patient was considered as one group in the fivefold cross-validation to ensure that all lesions for each patient were in the training/test set of a fold	3D slicer and 3D using the Pyradiomics package in Python (3.7)	Three models: each model was based on a Cox’s proportional hazards model.
Staal et al., 2021 [14]	Retrospective	CT	82 CRC patients with 127 LM treated by ablation	One cohort. Internal validation.	-	Kruskal–Wallis test was applied to evaluate whether the selected radiomics features were influenced by differences between scanners. Combined model yielded a c-statistic. Multivariable Cox regression
Reimer et al., 2018 [15]	Retrospective	MRI	37 CRLM patients treated by TARE	One cohort.	Mint Lesion ™ 3.0 (Mint Medical GmbH, Dossenheim, Germany)	Mann–Whitney *U* test. AUC and sensitivity and specificity were calculated.
Shuer et al., 2019 [16]	Retrospective	CT and MRI	102 CRLM treated by resection	One cohort.	Pyradiomics plugin to 3D Slicer	Cox regression coefficients
Ahm et al., 2016 [17]	Retrospective	CT including quadruple-phase (*n* = 27), triple-phase (*n* = 141), double-phase (*n* = 11) and single-phase CT (*n* = 54)	145 patients	Two cohorts. Validation cohorts (*n* = 90) and derivation cohorts (*n* = 145).	In-house software program (Medical Imaging Solution for Segmentation and Texture Analysis).	Student *t,* Mann–Whitney *U* test, ×2 or Fisher exact test. Multivariate logistic regression analysis.
Giannini et al., 2020 [18]	Included in HERACLES trial	CT	38 patients	Two cohorts. Training cohort 28 patients (108 lesions), validation cohort 10 patients (33 lesions).	Mipav software. In-house framework based on C++ and libraries	Genetic algorithms, algorithms belonging to the computational intelligence field.
Beckers et al., 2018 [19]	Retrospective	CT	70 CRLM patients	Two cohorts. 60 patients with chemotherapy and 10 patients without chemotherapy. No validation.	2D Texture analysis was performed with in-house software written in Python (MANGO; Multi-image Analysis GUI, Research Imaging Institute).	Shapiro–Wilk test was used to test for normality. Independent sample *t* tests. Multivariable Cox proportional hazards models
Andersen et al., 2019 [20]	Exploratory study	CT	27 CRLM patients treated by regorafenib	One cohort	-	-
Zhang et al., 2019 [21]	Retrospective	MRI	26 CRC patients with 193 LM	One cohort	MATLAB (MATLAB R2011b, MathWorks, Inc., Natick, MA, USA)	Student’s *t* test or Mann–Whitney *U* test when not normally distributed. Multivariable logistic regression analysis
Lubner et al., 2015 [22]	Retrospective	CT	77 CRLM patients	One cohort	TexRAD Ltd., (Somerset, UK)	Correlated using Cox proportional hazards models
Simpson et al., 2017 [23]	Retrospective	CT	198 patients	One cohort	Scout Liver (Pathfinder Technologies Inc., Nashville, TN, USA)	Kaplan–Meier and Cox proportional hazards models
Ganeshan et al., 2007 [24]	Retrospective	CT	27 patients	One cohort	TexRAD Ltd., (Somerset, UK) MATLAB (Mathworks Inc, Natick, MA, USA)	Cox regression analysis and the statistical significance of contingency tables was assessed using Fischer’s exact test.
Rahmim et al., 2019 [25]	Retrospective	FDG PET/CT	52 CRLM patients	One cohort	Hermes Hybrid Viewer PDR and MATLAB	Kaplan–Meier and Cox proportional hazards models
Dercle et al., 2020 [26]	Retrospective	CT	667 CRLM patients	Two cohorts. Randomly assigned (2:1) to training or validation sets. Predicted tumor sensitivity to treatment was measured using AUC in the validation sets of the four cohorts consisting of patients that were not used for training.	MATLAB (Mathworks, Natick, MA, USA)	Variance and v2 test were performed to compare categorical variables. Cox regression was used to investigate the effect of survival variables, and log-rank test was used to compare survival times of two groups.
Dohan et al., 2019 [27]	Multicenter prospective	CT	491 CRLM patients treated by FOLFIRI and bevacizumab	Two cohorts. Training cohort in 120 patients, and validate cohort in 110 patients. External validation was performed in another cohort of 40 patients	TexRAD Ltd., (Somerset, UK)	Multivariable Cox, Kaplan–Meier and log-rank
Ravanelli et al., 2019 [28]	Retrospective	CT	43 CRLM patients	Two cohorts. 23 treated with bevacizumab-containing chemotherapy (group A), and 20 with standard chemotherapy (group B)	MATLAB (Natick, MA, USA)	Multivariable logistic regression

CT: computed tomography; MRI: magnetic resonance imaging; FDG PET/CT: fluorodeoxyglucose positron emission tomography/computed tomography; CRC: colorectal carcinoma; LM: liver metastases; CRCLM: colorectal carcinoma liver metastases; HCC: hepatocellular carcinoma; HHs: hepatic hemangiomas; FLLs: focal liver lesions; AUC: area under curve; ROC: receiver operating characteristic.

## References

[1] Di Martino M., Rompianesi G., Mora-Guzmán I., Martín-Pérez E., Montalti R., Troisi R.I. (2020). Systematic review and meta-analysis of local ablative therapies for resectable colorectal liver metastases. Eur. J. Surg. Oncol..

[2] Valderrama-Treviño A.I., Barrera-Mera B., Ceballos-Villalva J.C., Montalvo-Javé E. (2017). Hepatic Metastasis from Colorectal Cancer. Euroasian J. Hepato-Gastroenterol..

[3] Siebenhüner A.R., Güller U., Warschkow R. (2020). Population-based SEER analysis of survival in colorectal cancer patients with or without resection of lung and liver metastases. BMC Cancer.

[4] Saini A., Breen I., Pershad Y., Naidu S., Knuttinen M.G., Alzubaidi S., Sheth R., Albadawi H., Kuo M., Oklu R. (2018). Radiogenomics and Radiomics in Liver Cancers. Diagnostics.

[5] Becker A.S., Schneider M.A., Wurnig M.C., Wagner M., Clavien P.A., Boss A. (2018). Radiomics of liver MRI predict metastases in mice. Eur. Radiol. Exp..

[6] Taghavi M., Trebeschi S., Simões R., Meek D.B., Beckers R.C.J., Lambregts D.M.J., Verhoef C., Houwers J.B., van der Heide U.A., Beets-Tan R.G.H. (2021). Machine learning-based analysis of CT radiomics model for prediction of colorectal metachronous liver metastases. Abdom. Radiol..

[7] Rao S.-X., Lambregts D., Schnerr R.S., van Ommen W., van Nijnatten T.J., Martens M.H., Heijnen L.A., Backes W.H., Verhoef C., Zeng M.-S. (2014). Whole-liver CT texture analysis in colorectal cancer: Does the presence of liver metastases affect the texture of the remaining liver?. United Eur. Gastroenterol. J..

[8] Liang M., Cai Z., Zhang H., Huang C., Meng Y., Zhao L., Li D., Ma X., Zhao X. (2019). Machine Learning-based Analysis of Rectal Cancer MRI Radiomics for Prediction of Metachronous Liver Metastasis. Acad. Radiol..

[9] Oyama A., Hiraoka Y., Obayashi I., Saikawa Y., Furui S., Shiraishi K., Kumagai S., Hayashi T., Kotoku J. (2019). Hepatic tumor classification using texture and topology analysis of non-contrast-enhanced three-dimensional T1-weighted MR images with a radiomics approach. Sci. Rep..

[10] Li Z., Mao Y., Huang W., Li H., Zhu J., Li W., Li B. (2017). Texture-based classification of different single liver lesion based on SPAIR T2W MRI images. BMC Med. Imaging.

[11] Jansen M.J.A., Kuijf H.J., Veldhuis W.B., Wessels F.J., Viergever M.A., Pluim J.P.W. (2019). Automatic classification of focal liver lesions based on MRI and risk factors. PLoS ONE.

[12] Gatos I., Tsantis S., Karamesini M., Spiliopoulos S., Karnabatidis D., Hazle J.D., Kagadis G.C. (2017). Focal liver lesions segmentation and classification in nonenhanced T2-weighted MRI. Med. Phys..

[13] Taghavi M., Staal F., Munoz F.G., Imani F., Meek D.B., Simões R., Klompenhouwer L.G., van der Heide U.A., Beets-Tan R.G.H., Maas M. (2021). CT-Based Radiomics Analysis Before Thermal Ablation to Predict Local Tumor Progression for Colorectal Liver Metastases. Cardiovasc. Interv. Radiol..

[14] Staal F., Taghavi M., van der Reijd D., Gomez F., Imani F., Klompenhouwer E., Meek D., Roberti S., de Boer M., Lambregts D. (2021). Predicting local tumour progression after ablation for colorectal liver metastases: CT-based radiomics of the ablation zone. Eur. J. Radiol..

[15] Reimer R.P., Reimer P., Mahnken A.H. (2018). Assessment of Therapy Response to Transarterial Radioembolization for Liver Metastases by Means of Post-treatment MRI-Based Texture Analysis. Cardiovasc. Interv. Radiol..

[16] Shur J., Orton M., Connor A., Fischer S., Moulton C.-A., Gallinger S., Koh D.-M., Jhaveri K.S. (2019). A clinical-radiomic model for improved prognostication of surgical candidates with colorectal liver metastases. J. Surg. Oncol..

[17] Ahn S.J., Kim J.H., Park S.J., Han J.K. (2016). Prediction of the therapeutic response after FOLFOX and FOLFIRI treatment for patients with liver metastasis from colorectal cancer using computerized CT texture analysis. Eur. J. Radiol..

[18] Giannini V., Rosati S., DeFeudis A., Balestra G., Vassallo L., Cappello G., Mazzetti S., de Mattia C., Rizzetto F., Torresin A. (2020). Radiomics predicts response of individual HER2 -amplified colorectal cancer liver metastases in patients treated with HER2 -targeted therapy. Int. J. Cancer.

[19] Beckers R.C.J., Trebeschi S., Maas M., Schnerr R.S., Sijmons J.M.L., Beets G.L., Houwers J.B., Beets-Tan R.G.H., Lambregts D.M.J. (2018). CT texture analysis in colorectal liver metastases and the surrounding liver parenchyma and its potential as an imaging biomarker of disease aggressiveness, response and survival. Eur. J. Radiol..

[20] Andersen I.R., Thorup K., Andersen M.B., Olesen R., Mortensen F.V., Nielsen D.T., Rasmussen F. (2019). Texture in the monitoring of regorafenib therapy in patients with colorectal liver metastases. Acta Radiol..

[21] Zhang H., Li W., Hu F., Sun Y., Hu T., Tong T. (2018). MR texture analysis: Potential imaging biomarker for predicting the chemotherapeutic response of patients with colorectal liver metastases. Abdom. Radiol..

[22] Lubner M.G., Stabo N., Lubner S.J., del Rio A.M., Song C., Halberg R.B., Pickhardt P.J. (2015). CT textural analysis of hepatic metastatic colorectal cancer: Pre-treatment tumor heterogeneity correlates with pathology and clinical outcomes. Abdom. Imaging.

[23] Simpson A.L., Doussot A., Creasy J.M., Adams L.B., Allen P.J., DeMatteo R.P., Gönen M., Kemeny N.E., Kingham T.P., Shia J. (2017). Computed Tomography Image Texture: A Noninvasive Prognostic Marker of Hepatic Recurrence After Hepatectomy for Metastatic Colorectal Cancer. Ann. Surg. Oncol..

[24] Ganeshan B., Miles K.A., Young R.C., Chatwin C.R. (2007). Hepatic Enhancement in Colorectal Cancer: Texture Analysis Correlates with Hepatic Hemodynamics and Patient Survival. Acad. Radiol..

[25] Rahmim A., Bak-Fredslund K.P., Ashrafinia S., Lu L., Schmidtlein C., Subramaniam R.M., Morsing A., Keiding S., Horsager J., Munk O.L. (2019). Prognostic modeling for patients with colorectal liver metastases incorporating FDG PET radiomic features. Eur. J. Radiol..

[26] Dercle L., Lu L., Schwartz L.H., Qian M., Tejpar S., Eggleton P., Zhao B., Piessevaux H. (2020). Radiomics Response Signature for Identification of Metastatic Colorectal Cancer Sensitive to Therapies Targeting EGFR Pathway. J. Natl. Cancer Inst..

[27] Dohan A., Gallix B., Guiu B., le Malicot K., Reinhold C., Soyer P., Bennouna J., Ghiringhelli F., Barbier E., Boige V. (2020). Early evaluation using a radiomic signature of unresectable hepatic metastases to predict outcome in patients with colorectal cancer treated with FOLFIRI and bevacizumab. Gut.

[28] Ravanelli M., Agazzi G.M., Tononcelli E., Roca E., Cabassa P., Baiocchi G.L., Berruti A., Maroldi R., Farina D. (2019). Texture features of colorectal liver metastases on pretreatment contrast-enhanced CT may predict response and prognosis in patients treated with bevacizumab-containing chemotherapy: A pilot study including comparison with standard chemotherapy. Radiol. Med..

[29] Fiz F., Viganò L., Gennaro N., Costa G., la Bella L., Boichuk A., Cavinato L., Sollini M., Politi L.S., Chiti A. (2020). Radiomics of Liver Metastases: A Systematic Review. Cancers.

[30] Langley R.R., Fidler I.J. (2011). The seed and soil hypothesis revisited-The role of tumor-stroma interactions in metastasis to different organs. Int. J. Cancer.

[31] Milette S., Sicklick J.K., Lowy A.M., Brodt P. (2017). Molecular Pathways: Targeting the Microenvironment of Liver Metastases. Clin. Cancer Res..

[32] Guo Y., Ji X., Liu J., Fan D., Zhou Q., Chen C., Wang W., Wang G., Wang H., Yuan W. (2019). Effects of exosomes on pre-metastatic niche formation in tumors. Mol. Cancer.

[33] Weidle U.H., Birzele F., Kollmorgen G., Rüger R. (2017). The Multiple Roles of Exosomes in Metastasis. Cancer Genom.-Proteom..

[34] Liu X., Li Y., Sun Z., Li S., Wang K., Fan X., Liu Y., Wang L., Wang Y., Jiang T. (2018). Molecular profiles of tumor contrast enhancement: A radiogenomic analysis in anaplastic gliomas. Cancer Med..

[35] Beig N., Bera K., Prasanna P., Antunes J., Correa R., Singh S., Bamashmos A.S., Ismail M., Braman N., Verma R. (2020). Radiogenomic-Based Survival Risk Stratification of Tumor Habitat on Gd-T1w MRI Is Associated with Biological Processes in Glioblastoma. Clin. Cancer Res..

[36] Liu P., Tan X.-Z., Zhang T., Gu Q.-B., Mao X.-H., Li Y.-C., He Y.-Q. (2021). Prediction of microvascular invasion in solitary hepatocellular carcinoma ≤ 5 cm based on computed tomography radiomics. World J. Gastroenterol..

[37] Li Y., Zhang Y., Fang Q., Zhang X., Hou P., Wu H., Wang X. (2021). Radiomics analysis of [18F]FDG PET/CT for microvascular invasion and prognosis prediction in very-early- and early-stage hepatocellular carcinoma. Eur. J. Nucl. Med. Mol. Imaging.

[38] Moro C.F., Bozóky B., Gerling M. (2018). Growth patterns of colorectal cancer liver metastases and their impact on prognosis: A systematic review. BMJ Open Gastroenterol..

[39] Van Dam P.-J., van der Stok E.P., Teuwen L.-A., van den Eynden G.G., Illemann M., Frentzas S., Majeed A.W., Eefsen R.L., van den Braak R.R.J.C., Lazaris A. (2017). International consensus guidelines for scoring the histopathological growth patterns of liver metastasis. Br. J. Cancer.

[40] Beig N., Singh S., Bera K., Prasanna P., Singh G., Chen J., Saeed Bamashmos A., Barnett A., Hunter K., Statsevych V. (2021). Sexually dimorphic radiogenomic models identify distinct imaging and biological pathways that are prognostic of overall survival in glioblastoma. Neuro-oncology.

[41] Qian Z., Li Y., Sun Z., Fan X., Xu K., Wang K., Li S., Zhang Z., Jiang T., Liu X. (2018). Radiogenomics of lower-grade gliomas: A radiomic signature as a biological surrogate for survival prediction. Aging.

[42] Lin P., Wen D.-Y., Chen L., Li X., Li S.-H., Yan H.-B., He R.-Q., Chen G., He Y., Yang H. (2020). A radiogenomics signature for predicting the clinical outcome of bladder urothelial carcinoma. Eur. Radiol..

[43] Yin Q., Hung S.-C., Wang L., Lin W., Fielding J.R., Rathmell W.K., Khandani A.H., Woods M.E., Milowsky M.I., Brooks S.A. (2017). Associations between Tumor Vascularity, Vascular Endothelial Growth Factor Expression and PET/MRI Radiomic Signatures in Primary Clear-Cell–Renal-Cell-Carcinoma: Proof-of-Concept Study. Sci. Rep..

[44] Arefan D., Hausler R.M., Sumkin J.H., Sun M., Wu S. (2021). Predicting cell invasion in breast tumor microenvironment from radiological imaging phenotypes. BMC Cancer.

[45] Lee J.Y., Lee K.-S., Seo B.K., Cho K.R., Woo O.H., Song S.E., Kim E.-K., Lee H.Y., Kim J.S., Cha J. (2021). Radiomic machine learning for predicting prognostic biomarkers and molecular subtypes of breast cancer using tumor heterogeneity and angiogenesis properties on MRI. Eur. Radiol..

[46] Xiao J., Rahbar H., Hippe D.S., Rendi M.H., Parker E.U., Shekar N., Hirano M., Cheung K.J., Partridge S.C. (2021). Dynamic contrast-enhanced breast MRI features correlate with invasive breast cancer angiogenesis. NPJ Breast Cancer.

[47] Smedley N.F., Aberle D.R., Hsu W. (2021). Using deep neural networks and interpretability methods to identify gene expression patterns that predict radiomic features and histology in non-small cell lung cancer. J. Med Imaging.

[48] Jeon S.H., Lim Y.J., Koh J., Chang W.I., Kim S., Kim K., Chie E.K. (2021). A radiomic signature model to predict the chemoradiation-induced alteration in tumor-infiltrating CD8+ cells in locally advanced rectal cancer. Radiother. Oncol..

[49] Sun R., Limkin E.J., Vakalopoulou M., Dercle L., Champiat S., Han S.R., Verlingue L., Brandao D., Lancia A., Ammari S. (2018). A radiomics approach to assess tumour-infiltrating CD8 cells and response to anti-PD-1 or anti-PD-L1 immunotherapy: An imaging biomarker, retrospective multicohort study. Lancet Oncol..

[50] Mu W., Jiang L., Shi Y., Tunali I., Gray J.E., Katsoulakis E., Tian J., Gillies R.J., Schabath M.B. (2021). Non-invasive measurement of PD-L1 status and prediction of immunotherapy response using deep learning of PET/CT images. J. Immunother. Cancer.

[51] Zhang J., Wu Z., Zhang X., Liu S., Zhao J., Yuan F., Shi Y., Song B. (2020). Machine learning: An approach to preoperatively predict PD-1/PD-L1 expression and outcome in intrahepatic cholangiocarcinoma using MRI biomarkers. ESMO Open.

[52] Yu Y., He Z., Ouyang J., Tan Y., Chen Y., Gu Y., Mao L., Ren W., Wang J., Lin L. (2021). Magnetic resonance imaging radiomics predicts preoperative axillary lymph node metastasis to support surgical decisions and is associated with tumor microenvironment in invasive breast cancer: A machine learning, multicenter study. EBioMedicine.

[53] Starosolski Z., Courtney A.N., Srivastava M., Guo L., Stupin I., Metelitsa L.S., Annapragada A., Ghaghada K.B. (2021). A Nanoradiomics Approach for Differentiation of Tumors Based on Tumor-Associated Macrophage Burden. Contrast Media Mol. Imaging.

[54] Siveen K.S., Raza A., Ahmed E.I., Khan A.Q., Prabhu K.S., Kuttikrishnan S., Mateo J.M., Zayed H., Rasul K., Azizi F. (2019). The Role of Extracellular Vesicles as Modulators of the Tumor Microenvironment, Metastasis and Drug Resistance in Colorectal Cancer. Cancers.

[55] Lugini L., Valtieri M., Federici C., Cecchetti S., Meschini S., Condello M., Signore M., Fais S. (2016). Exosomes from human colorectal cancer induce a tumor-like behavior in colonic mesenchymal stromal cells. Oncotarget.

[56] Engstrand J., Nilsson H., Strömberg C., Jonas E., Freedman J. (2018). Colorectal cancer liver metastases—A population-based study on incidence, management and survival. BMC Cancer.

[57] Liu Y., Cao X. (2016). Characteristics and Significance of the Pre-metastatic Niche. Cancer Cell.

[58] Van den Eynden G.G., Majeed A.W., Illemann M., Vermeulen P.B., Bird N.C., Høyer-Hansen G., Eefsen R.L., Reynolds A.R., Brodt P. (2013). The Multifaceted Role of the Microenvironment in Liver Metastasis: Biology and Clinical Implications. Cancer Res..

[59] Rudmik L.R., Magliocco A.M. (2005). Molecular mechanisms of hepatic metastasis in colorectal cancer. J. Surg. Oncol..

[60] Han Y., Chai F., Wei J., Yue Y., Cheng J., Gu D., Zhang Y., Tong T., Sheng W., Hong N. (2020). Identification of Predominant Histopathological Growth Patterns of Colorectal Liver Metastasis by Multi-Habitat and Multi-Sequence Based Radiomics Analysis. Front. Oncol..

[61] Hadrup S.R., Donia M., Straten P.T. (2012). Effector CD4 and CD8 T Cells and Their Role in the Tumor Microenvironment. Cancer Microenviron..

[62] Rubie C., Oliveira V., Kempf K., Wagner M., Tilton B., Rau B., Kruse B., König J., Schilling M. (2006). Involvement of Chemokine Receptor CCR6 in Colorectal Cancer Metastasis. Tumor Biol..

[63] Wagner H.E., Toth C.A., Steele G.D., Thomas P. (1992). Metastatic potential of human colon cancer cell lines: Relationship to cellular differentiation and carcinoembryonic antigen production. Clin. Exp. Metastasis.

[64] Böckelman C., Engelmann B.E., Kaprio T., Hansen T.F., Glimelius B. (2014). Risk of recurrence in patients with colon cancer stage II and III: A systematic review and meta-analysis of recent literature. Acta Oncol..

[65] Grothey A., Sobrero A.F., Shields A.F., Yoshino T., Paul J., Taieb J., Souglakos J., Shi Q., Kerr R., Labianca R. (2018). Duration of Adjuvant Chemotherapy for Stage III Colon Cancer. N. Engl. J. Med..

[66] Paget S. (1989). The distribution of secondary growths in cancer of the breast.1889. Cancer Metastasis Rev..

[67] Creasy J.M., Cunanan K.M., Chakraborty J., McAuliffe J.C., Chou J., Gonen M., Ba V.S.K., Weiser M.R., Balachandran V.P., Drebin J.A. (2021). Differences in Liver Parenchyma are Measurable with CT Radiomics at Initial Colon Resection in Patients that Develop Hepatic Metastases from Stage II/III Colon Cancer. Ann. Surg. Oncol..

[68] Fiz F., Costa G., Gennaro N., la Bella L., Boichuk A., Sollini M., Politi L., Balzarini L., Torzilli G., Chiti A. (2021). Contrast Administration Impacts CT-Based Radiomics of Colorectal Liver Metastases and Non-Tumoral Liver Parenchyma Revealing the “Radiological” Tumour Microenvironment. Diagnostics.

[69] Rizzetto F., Calderoni F., de Mattia C., Defeudis A., Giannini V., Mazzetti S., Vassallo L., Ghezzi S., Sartore-Bianchi A., Marsoni S. (2020). Impact of inter-reader contouring variability on textural radiomics of colorectal liver metastases. Eur. Radiol. Exp..

[70] Granata V., Fusco R., Barretta M.L., Picone C., Avallone A., Belli A., Patrone R., Ferrante M., Cozzi D., Grassi R. (2021). Radiomics in hepatic metastasis by colorectal cancer. Infect. Agents Cancer.

[71] Zwanenburg A., Vallières M., Abdalah M.A., Aerts H.J.W.L., Andrearczyk V., Apte A., Ashrafinia S., Bakas S., Beukinga R.J., Boellaard R. (2020). The Image Biomarker Standardization Initiative: Standardized Quantitative Radiomics for High-Throughput Image-based Phenotyping. Radiology.

